# Experiential meaning as meaning making choice in article writing: A case study of female and male writers

**DOI:** 10.1016/j.heliyon.2021.e06909

**Published:** 2021-04-28

**Authors:** Rosi Anjarwati, Slamet Setiawan, Kisyani Laksono

**Affiliations:** a Universitas Negeri Surabaya, STKIP PGRI Jombang, Indonesia; b Universitas Negeri Surabaya, Indonesia

**Keywords:** Experiential meaning, Meaning-making choice, Research article, Writing

## Abstract

Meaning-making choice place a significant position in written communication, as indirect contact needs a particular strategy to achieve the objective. This case study explores how female and male writers utilized their meaning-making choice through experiential meaning in their introduction part of journal article writing published in JEELL. Research articles written by five female writers and five male writers with English teachers' professional backgrounds are involved in this study. The results indicate that both female and male writers tend to use a particular process, such as material processes, to represent their real-world experiences of *doing* and *happening*. However, it is also found that there is a difference in the technique used in expressing material processes by female and male writers. The study also implies that male writers apply more verbal processes than female writers in citing or synthesizing sources. Furthermore, the investigations reveal that male writers produced more various type of mental processes than female writers.

## 1 Introduction

Writing in an academic setting needs some considerations; one of them is meaning-making construction. Bird (2010) believes that meaning-making should be produced through writing to give both writer and reader involvement in verbal culture; that condition might be absent in writing as non-verbal communication. Yasuda (2015) mentioned that meaning-making choice is created to accomplish a particular rhetorical objective through writing. Moreover, Byrnes (2013) also states the importance of teaching meaning in writing since writing is about meaning-making. Hence, a meaning-making choice can be defined as the writer's ability to construct meaning through certain words in achieving the specific linguistic objective. In choosing those words, grammar always becomes a central consideration.

As part of productive skills, meaning-making in writing has some distinctive features compared to meaning-making in speaking. These distinctions refer to the typical condition of language use and the linguistic implications of mode (Eggins, 2004). The characteristic of written discourse tends to be less interactive, not face-to-face, using language to reflect, not spontaneous, and using formal and particular circumstances compared to spoken discourse. Besides, the linguistic implications of mode in written language tend to be monologic organization, independent context, synoptic structure, tend to use ‘prestige’ lexis and standard grammar with more simplicity, and dense lexical. Thus, meaning-making in writing has unique features compared to other modalities.

From the Systemic Functional Linguistics (SFL) point of view, grammar is not only considered as a language system or rule; it involves meaning realized through metafunctions. As Bloor and Bloor (2004) stated, grammar in the SFL perspective is viewed as a study of how word choice, grammatical resource, and other linguistic forms create meaning. In using a language to produce specific meanings, people need to consider a particular situation involved in communication and some aspects related to the situation. Besides, Halliday & Matthiessen (2014) mention three different types of meaning known as metafunction, namely textual meaning, interpersonal meaning, and experiential (ideational) meaning. Among those three meanings, the experiential meaning is defined as the meaning of a clause which portrays some process in human experience.

Experiential meaning can be expressed through various writing genres, including journal articles. Journal article has become popular academic writing nowadays due to the advance of science and technology and the demand for publishing at a higher educational level. Writing a scientific article requires particular language use, such as typically complicated terminology, generally used of the passive construction, and a specific referencing system. Moreover, Hartley (2008) states that scientific writing should be accurate, detached, and neutral. Thus, the expressed meaning needs to have the clarity to achieve the writer's intention.

There are several parts of the scientific articles as an academic writing product. The scientific article has a typical format such as: abstract, introduction, method, result and discussion, and conclusion (Burgess and Cargill, 2013); each part carries out a different function. The introduction section of a research article usually plays a crucial role in which it should attract the reader's attention and give an overview of the entire article. As stated by Wallwork (2011), the research article writer requires to provide background knowledge to the readers in the introduction section to make them understand the importance of the research finding and the novelty of the research. Consequently, the writer should convince the readers that his idea in doing the research is worth-conducted.

Many researchers hold the view that there is a considerable number of aspects that may have an impact on writing; for instance, the writer's social class, education, marital status, and gender. Among those socio-cultural characteristics, gender has become a crucial issue to be discussed in writing. Although many studies about gender differences in writing have been conducted (Awan and Azeem, 2017; Jones and Myhill, 2007; Lee, 2013), the findings consistently indicate that gender difference with younger age is more obvious. Therefore, this research aimed at exploring whether gender differences influence adult writers' performance. There was an assumption that females tend to express something more freely, emotionally, and insistently than males in some cases. Ishikawa (2015) strengthens this view by showing that female writers in her study apply more particular words in their writing concerning cognitive psychological processes and personal pronoun. Additionally, female writers indicate their care to others and attempt to reduce the effect of their argument in writing. On the other hand, male writers tend to utilize more nouns related to the topic with great attention to things or events' specific features.

The previous research has highlighted gender difference in Foreign Language writing context; Lahuerta (2020) investigated written language accuracy of essays produced by Content and Language Integrated Learning (CLIL) and non-CLIL students involving grade and gender. The study revealed that male students created significantly more mistakes compared to female and females constructed considerably fewer syntactic error. A similar study about kinds of written error based on gender also carried out by Boroomand and Rostami Abusaeedi (2013). Other studies focused on the effect of gender on linguistic features in writing letter (Hamdi asl & Dabaghi, 2012) and related it to writing strategy (Bai et al., 2020). In the Indonesia context, gender and writing have been explored by some researchers. Suganob-Nicolau and Sukamto (2016) investigated complex sentences produced by different gender in writing narrative text, while Pasaribu (2017) focused on metadiscourse markers in academic essays written by female and male students. Furthermore, the study that related to gender difference with motivation and writing proficiency have also been conducted (Cahyono and Rahayu, 2020). Thus, there is a need for further investigation of gender and writing in term of meaning-making choice with different writing product.

This current research focused on the experiential meaning as a meaning-making choice produced by writers of different gender through their article writing. Using certain types of lexicogrammatical features in writing an introduction of a journal article that is created by distinctive gender will enable us to understand how the writers convince the readers that their research is essential to be done. Moreover, it can be beneficial to know whether female and male writers managed their background of the study by using a particular technique of expressing experiential meaning in writing. This research aims to reveal the experiential meaning used as a meaning-making choice by female and male writers from the Indonesian context in the introduction part of the journal article. Furthermore, the writer intended to trace the pattern of sentences used by female and male writers through the Transitivity system analysis to achieve the goal of writing a journal article. The analysis result will contribute to a comprehensive view of how female and male writers constructed reality through their article writing.

## 2 Literature review

### 2.1 Experiential meaning

Experiential meaning is one of the metafunctions in Systemic Functional Linguistics (SFL). Systemic Functional Linguistic was developed by Halliday in 1960s which considers language from the functional usage and famous for being called metafunctions; it means that the utterance produced is based on what needs to be revealed by the speaker or writer (Ong'onda, 2016). It regards grammar as a source of meaning-making and claims the relationship between form and meaning. Thus, there are three types of meanings in the SFL point of view; textual meaning that produces the organization of the text, interpersonal meaning which creates and preserves social relationship, and Experiential meaning that considers the clause as a representation of both ‘outer ‘and ‘inner’ experiences.

Several experts and researchers have proposed some definitions of experiential meaning. Bloor and Bloor (2004) stated that experiential meaning refers to the expression and form of how the world is viewed based on our awareness through language. According to Mehmood et al. (2014), experiential meaning functions as the manifestation of the real-world experience in which the occurrence or thing occurs, and people or other actors do something, as well as inner world experience that is going on inside in the world of consciousness. Concerning this definition, Eggins (2004) added that experiential meaning is conveyed by using the Transitivity system. The system choices would be linked to the Field factor, with the selection of process types and participant roles seen as the encoding of their experiential reality by interactants, namely: the environment of acts, relationships, participants and circumstances that offer their speech content. It can be concluded that experiential meaning is a type of meaning as the expression of reality through language, which is realized by process type, participant involved in the process, and circumstance around the process.

Six types of processes are the categories of verbs involved in transitivity system analysis. Those are material processes, mental processes, behavioral processes, verbal processes, existential processes, and relational processes (Eggins, 2004; Halliday and Matthiessen, 2014). In construing the act of *doing* and the *happening* meaning, the material process is needed, while the mental process carries out the meaning of *sensing.* These two kinds of processes can be differentiated from the system of tense. The behavioral process has the meaning of *behaving* that can express both physical and psychological activities. The meaning of *saying* is presented by the verbal process, while the meaning of *existing* is conveyed by the existential process. The last category is named relational process, whose meaning is *being.* Each process type is realized by particular verbs which have different function as mentioned by Gwilliams and Fontaine (2015) that the syntax and semantics of the component (verb and participant) are the fundamental consideration of the process classification. The more detailed process types and participants as characterized in Systemic Functional Linguistics are presented in Table 1.

**Table 1:** Process types, their meanings, and characteristic participants.

Process type	Category meaning	Participants, directly involved	Participants, obliquely involved
material:	‘doing’	Actor, Goal	Recipient, Client; Scope; Initiator; Attribute
action	‘doing’		
event	‘happening’		
behavioral	‘behaving’	Behaver	Behavior
mental:	‘sensing’	Senser, Phenomenon	Inducer
perception	‘seeing’		
cognition	‘thinking’		
desideration	‘wanting’		
emotion	‘feeling’		
Verbal	‘saying’	Sayer, Target	Receiver; Verbiage
relational:	‘being’		
attribution	‘attributing’	Carrier, Attribute	Attributor; Beneficiary
identification	‘identifying’	Identified, Identifier; Token, Value	Assigner
Existential	‘existing’	Existent	

Adopted from: (Halliday and Matthiessen, 2014, p. 311, p. 311).

Research about experiential meaning through transitivity analysis has been applied in various fields. For instance, Kondowe (2014) has investigated the president and ideologies through his inaugural address by using transitivity analysis; it showed that Halliday's transitivity concept could be beneficial in exploring language and politics. The second study has pointed out the used of transitivity system in psychology; here, the researchers aimed at proposing a framework to investigate the social value, particularly in the term of point of view which carried various style and meaning (Tsirogianni and Sammut, 2014). Another study also indicated the implementation of transitivity framework in ESP (medical-dental) which focused on the analysis of rhetorical moves in dental research articles abstracts by comparing Thai written journal and international journal (Vathanalaoha and Tangkiengsirisin, 2018). While the implementation of transitivity analysis in education and language was demonstrated in Munalim's (2017) study that had explored mental processes produced by teachers in writing their reflection papers. The findings reveal the type of participant in a mental process called a Phenomenon to manifest teachers' inner world occurrence. There is an essential piece of information for creating a better understanding of how mental process usage influences teachers' points of views about their profession and deals with it. Another study is conducted by Hermawan and Rahyono (2019) in which they analyzed the experiential meaning and interpersonal position of readers in the science textbook. The results revealed that the majority of pictures used in the textbook represented human perception about the real object while the position of the readers is placed as receivers of the message from a more powerful person in the interpersonal meaning analysis.

### 2.2 Meaning-making choice in writing

Meaning-making choice plays an essential part in communication, both in spoken and written. It has been defined from several distinctive points of view; Halliday and Matthiessen (2014) view meaning-making as the function of grammar that explains grammatical classification by indicating its meaning. It is related to semantic sophistication, which requires experiential, interpersonal, and textual meanings to be combined into linguistic units; it is possible because language is a semiotic system, a traditional coding system, arranged as a series of choices. Besides, Eggins (2004) explains that a semiotic understanding of the language system helps one understand the exploitation or inappropriateness of various linguistic choices to their meaning of use; and see language as a resource that is used to make sense in contexts. Thus, the meaning-making choice here deals with the perception of grammar as a whole language package, including words, sentences, and the process of making interpretations based on a particular context.

Systemic Functional Linguistics perspective enables linguists and educators to analyze grammar in writing as a procedure of making meaning rather than the mechanical procedure of word-formation. As stated by Halliday and Matthiessen (2014, p. 3), language is a resource for making meaning; therefore, the text is a process of making meaning. Furthermore, by choosing from similar meaning-making resources, every text may obtain its meaning. The traits and their selection are usually represented by the word “choice” as an approach to show the choice relationship. The writer's meaning-making is different from readers' meaning-making; the reader's meaning-making refers to the interpretive process of reading a text, which is directed by the reader's linguistic experiential storage device as he reads, interprets, and re-interprets different signs and patterns of text communication. On the other hand, the writer's meaning-making is defined as the writer's ability in organizing their meaning and choosing specific words based on the context (Byrnes, 2011).

For many years, study about writing focuses on error analysis (Doolan, 2017; Sermsook et al., 2017), genre (Emilia and Hamied, 2015; Nagao, 2019), and feedback in writing (Chen et al., 2011) while meaning-making choice in writing is still rare to be investigated. Meaning-making, which is developing from a Systemic Functional Linguistics point of view gives a new trend in analyzing writing products. Recently, Xuan (2018) utilized the framework of SFL in exploring adult Chinese L2 writing as meaning-making. The findings of the research reveals that there are registered variations in process type implementation of various writing tasks. Moreover, the process types used in their writing are plain and congruent.

Studies about meaning-making not only involved adolescent writer as the participant. Yasuda (2019) has researched to explore young writer's meaning-making choice in utilizing cohesive devices and interpersonal resources in the entirety text level. Since the study about young writer's meaning-making choice is rarely executed, the result of the study becomes attractive to be discussed mainly in interpersonal resource usage. Another result of research suggests applying Halliday's framework of grammar as a resource of meaning-making in teaching grammar in the L1 classroom (Myhill, 2018). From Myhill's study, it can be inferred that teaching grammar through writing is not merely about the language rules and tense used but also the linguistic choice students have made in creating particular meaning.

### 2.3 Gender difference in writing

Gender has become great attention in linguistics for a few decades as it is an exciting phenomenon in society that can affect how to use language in communication. Many people use the term of gender similar to sex; in fact, these two terminologies are different. According to Pryzgoda and Chrisler (2000) in Bijami (2013), gender denotes men's and women's behavior, social, and mental characteristics. Similarly, Kamari et al. (2012) point out gender as an acultural and community point of view about the social and circumstantial beliefs of men and women in a certain society. It has been shown that gender is not always about biological features as male or female; in fact, gender difference also reveals distinctive performance, which might appear in communication features.

There is a common belief that males and females utilize language differently, both in spoken and written form. Female is assumed to be more talkative than male in spoken language, yet Mahmud (2010) in her research, reveals that female students favor stating their opinion through writing while male students view speaking as a helpful way of communication. Another study is carried out by Suganob-Nicolau and Sukamto (2016); through their study, it was found that male students used more frequent simple sentences than female students. In addition, female students also demonstrated compound and complex sentences with more influential minds and inspiration. Nevertheless, male students produced more various lexis.

Gender might be the potential source that affects the language used, particularly in writing aspects. Adams and Simmons (2019) state that females managed to create a longer written composition with better quality than males. Equally, Babayiğit (2015) and Al-Saadi (2020) also reveals that female writers tend to outperform male writers in writing fluency, general quality, and lexis; male writers only perform better in the quality of writing organization. The parallel result was shown by Kim et al. (2015) in which females achieve higher writing scores viewed from writing dimensions such as spelling, handwriting automaticity, etc. Even though those studies explored both writing output and quality, there is no information on gender difference analyzed from Systemic Functional Linguistics, especially in terms of experiential meaning.

To date, different genders in writing have not been explored by using transitivity analysis. Nevertheless, a study about gender and language function in a written text which applied Halliday's framework of styles of writing proposed that female writers tend to used *involve* style; on the other hand, male incline to employ *informative* one (Alkhrisheh et al., 2019). *Involve* style refers to the writer's assumption that the readers have enough background knowledge so that they are required to be involved in the female writers' point of view. While *informative* style described as the use of more detailed information since male writers assumed that the readers should be given background knowledge.

## 3 Method

A case study is used in the current research to explore the meaning-making choice in the form of experiential meaning expressed in article writing produced by female and male writers. Creswell (2007) states that a case study is a method that comprises an issue explored through one or more cases in a certain system (e.g. a setting, a context). In this case, research articles are written by different gender of writers on the Journal of English Department of STKIP PGRI Jombang (JEELL) be the source of data.

### 3.1 Participants

The article writers in this study were chosen deliberately based on background affiliation as teacher and lecturer. Five female and five male writers were chosen from five different volumes during the 2017 to 2019 journal publication period. They are English teachers and lecturers from some regions in Indonesia who have more than three years of teaching experience with an age range between 30 and 45 years old. There is no specific information about their language proficiency level but they have experience in publishing their article before. Other socio-cultural aspects such as educational background, marital status, religion are sorted by the researcher before selecting the research article to ensure that those do not affect the writers' linguistics performance.

### 3.2 Data collection

The data of this research was obtained by using the documentation technique, the researchers took the final product of article writing published in JEELL by considering the writers' gender and affiliation background. The text was produced by the writers to be published in the Journal that went through the reviewing process. There was no specific topic of the article that became the consideration of selecting the text (article); each participant wrote a different topic related to language teaching. Particularly, the introduction section of the existing research article was analyzed by using the Transitivity system to explore experiential meaning produced as a meaning-making choice. The introduction section was chosen since the writers might elaborate their background of doing the research which has not been explored by using Transitivity.

### 3.3 Data analysis

The analysis model is adopted from the classification of processes proposed by Halliday and Matthiessen (2014) that consist of six types of process: material process, behavioral process, mental process, verbal process, relational process, and existential process. The analysis tool model is developed by adapting the work of Al-Janabi (2013) and Nguyen (2012) in which the complex sentence and compound-complex sentence are broken down into simple clauses to identify the process (verb) and participant involved in the process.

The introduction section was classified into sentences which then were classified into the types of sentence namely simple sentence, compound sentence, complex sentence, and compound-complex sentence. There were 196 sentences produced by female writers and 199 sentences created by male writers. The analysis was accomplished by investigating the type of process (verbs) and its function as well as the type of participant and circumstance in each clause. Prior to organizing the research, the ethical clearance confirmation from the Research Ethics Committee, STKIP PGRI Jombang was obtained as one of the requirements.

## 4 Results and discussion

### 4.1 Results

Experiential meanings expressed in research article writing are realized by the type of process, the participants involved, and circumstantial elements. In this study, the results are presented based on the gender of the research article writers. Each element of the experiential meanings is analyzed in the following section. The core of experiential meanings is construed by the process used in the clauses produced by the writers. Among six types of processes, it was found that all types are used in female writers' articles. It was demonstrated in the following table.

Table 2 shows the variation of process type applied in article writing produced by female writers in which the material process dominates the clauses in the introduction part. It expresses the writers' material world experience that functions to represent the meaning of doing and happening. As illustrated in example (a), it involved two participants, namely Actor (*They*) and Goal (*the material*). Since it demonstrates the meaning of doing, which was led to the material as the Goal, it belongs to the transitive material clause. Written in operative (active) form, this clause also describes a transformative clause because the outcome (Goal) has been changed as the effect of something done by the actor. Furthermore, the outcome in the example is explained more by the circumstance of enhancement especially *cause* indicating the purpose of the Goal as a transformation clause's feature. Actor and Goal are not the only participant types involved in material clauses created by female writers; Scope is also used. Unlike Goal, Scope is not affected directly by the process performed by Actor. It can be an object that exists regardless of the process which reveals the domain of the process; Scope can also express non-living things that indicate the process. One of the examples is expressed by the clause “*Sometimes, the teacher did the activity in the group of four to six…*” (clause F.2.52). In this clause, *the activity* is called Scope; it denotes the range of what teachers did.

**Table 2:** Process type produced by female writers.

No.	Process type	Frequency	Example
1.	Material process	262	*a. They won't use the material for communication as well.* (Clause F.2.39)
2.	Relational process	134	*b. In other words, feedback is a crucial part of the learning process.* (Clause F.1.10)
3.	Verbal process	27	*c. Some linguist stated that the teachers could be as effective as theories in the solution to the problem on foreign language teaching.* (Clause F.2.57)
4.	Mental process	22	*d. …they think that grammar is difficult.* (Clause F.2.33)
5.	Existential process	8	*e. There are some principles of the eclectic method.* (Clause F.2.70)
6.	Behavioural process	4	*f. Moreover, some students looked sleepy and bored.* (Clause F.2.51)

The second dominant process created is relational process; it can be found in one hundred and thirty-four clauses. Among six sub-categories of relational process, *intensive attributive* and *possessive attributive* are realized in females' article writing. In majority, intensive attributive is often used to state relationship requirement, such as in clause (b); here, the specification of relationship shows the quality of the thing (the first participant) called *Carrier* (*feedback*) which is introduced by quality attribute in the form of the adjective (*crucial*). In this context, it functions to convince readers about the importance of feedback in the learning process. Secondly, possessive attributive appears twenty-one times in the introduction part. One notable example is shown in the clause “*In other words, teacher's feedback has a fundamental role in realizing learning objective*.” (clause F.1.6). From the example, it can be inferred that the possession function as a process in which the *fundamental role’* ownership placed as an attribute assigned to the *teacher's feedback*. Indicating by the process (verb) of *has*, this clause signifies the Carrier (*teacher's feedback*) as the possessor and the attribute (*fundamental role*) as the possessed.

The third dominant process is verbal process, which is used in twenty-seven clauses. These clauses are commonly used to present quotations and reports; in this case, the female writers utilized it to cite some statements from scholars related to their research. The example is given in Table 2 clause (c) in which it is used to signify a proposition in the form of a report expresses semiosis meaning (neutral quoting). Involving two participants namely the Sayer (*some linguist*) that represents the speaker or writer and the Verbiage which functions to relate to what is said, the clause is operated to assert the writer's stance and persuade the readers to follow her point of view. In this context, the verbal clause serves as the supporting arguments to strengthen the writer's idea.

The mental process is used twenty-two times in three various types, namely cognitive, desiderative, and perceptive mental clauses. The cognitive mental process dominates the frequency with fifteen times used; one of them is expressed through clause (d). The example demonstrates meta-phenomenal in which the Senser (*they*) conveys a proposition of idea as a fact indicates by the conjunction *that* as a projected clause. This clause deals with experience related to cognition or thinking that is generally performed to state opinion. The second variant is labeled as a desiderative mental process which can be found in six clauses. To take the most striking example, the clause “*Some students do not like to speak in the classroom*.” (clause F.3.140). Introduced by the verb *like*, the clause expresses the meaning of desire for something in negation form. Furthermore, it states the directionality of ‘like-type’ that is used by the writer to illustrate the situation in the classroom related to her research concept. In addition to the two variants of mental process, the only clause that contains a perceptive mental clause is “*However, as the researcher has seen directly…”* (Clause F.3.123). Through this clause, the writer tries to interpret or show that she is aware of the research article's issue by showing empirical evidence. This empirical evidence is communicated from her perception that is implied by the verb *seen.*

The existential process that functioned to signify that something occurs and exists has been used in eight clauses. As presented in clause (e), the process is signified by the use of *there* followed by *be,* and a statement called existent functioned as participants. The word *there* in this clause has no representative function; it denotes the character of existence. The writer creates this clause to introduce a phenomenon about the eclectic method which becomes the concept in her research article.

Besides the five types of the process presented earlier, the behavioral process was found in four clauses. Clause in (f) shows the behavior that symbolizes the psychological process of consciousness. It exemplifies the meaning near to the mental process but is realized in the form of behavior. The writer uses the clause to depict the classroom situation that encourages her to research by applying particular teaching strategies.

The circumstance is another essential element of transitivity in revealing experiential meanings; Table 3 summarizes circumstantial function and its types utilized by female writers. Enhancing is the first function of circumstantial element applied; it is realized into four types i.e. *location, manner, cause*, and *extent*. Enhancing function in the form of location is demonstrated in (g); from the example, the writer applies location showing time which is placed at the beginning of the clause. The absolute temporal circumstance provides additional information to readers about the exact time of the material process that happened. The second is *manner* indicating quality that is expressed in (h). Here, the circumstance of quality symbolizes positive interpersonal evaluation introduced by the adverb of manner *well*. It answers the question of *how* that portrays the mental process' feature. The third is *cause* showing behalf as stated in (i); in this context, the writer completes the clause with the info for whose the action expressed through the material process is directed. It is different from the participant involved in the material process (Goal) in which this circumstantial element is not inbuilt in the clause, but a preposition followed by the person. The last is clause (j) called as extent showing duration. Indicated by a definite quantifier, this circumstance informs the readers about the material process's temporal interval in the form of a nominal group.

**Table 3:** Circumstance type produced by female writers.

No.	Circumstance Function	Circumstance type	Frequency	Example
1.	Enhancing	1) Location	58	*g.****In 2015****, Chaves and Guapacha made an eclectic professional development proposal for teaching language teachers. (Clause F.2.84)*
		2) Manner	33	*h. They will remember the pattern****well****but only in the short term memory. (Clause F.2.38)*
		3) Cause	19	*i. This section will list a few possible activities****for EFL teachers****… (Clause F.5.192)*
		4) Extent	3	*j. It is taught just****two hours for each week****. (Clause F.4.158)*
2.	Projecting	1) Matter	62	*k. …the purpose of speaking here may be to express opinion, to persuade someone****about something****…(Clause F.3.94)*
		2) Angle	2	***l. According to Brown****(2001, p.270), there are some features that make speaking as difficult language skill. (Clause F.3.110)*
3.	Extending	Accompaniment	11	*m. Informal speaking is used to make a communication****with their friends****…(Clause F.3.103)*
4.	Elaborating	Role	8	*n. …Sutanto (2015) remarks feedback****as the soul of learning experience in thesis writing****. (Clause F.1.4)*

The second function of circumstance found in female writers' article is projecting; it involves two sub-categories, namely *matter* and *angle*. The *matter* circumstance type is dominantly used and realized in sixty-two clauses. One example is (k) in which it projects the material clause and states what is described. Using preposition *about*, the circumstance answers concerning the subject of the process implied in the clause. While matter dominates the projecting function, angle indicating source only used two times. As demonstrated in (l), the *angle* functions to denote the information which presents someone's perspective through the clause. In this context, the complex preposition *according to* is applied to cite an expert's idea to strengthen the writer's claim toward *speaking* as the focus of her research.

Extending function becomes the third circumstance function applied by female writers. Accomplished by *accompaniment* type, eleven clauses signify the extending function such as in (m). What this example clearly illustrates is, it expresses a positive comitative that carries a meaning of *accompanied by*. Moreover, it symbolizes the process as a particular case of a process with one of the two creatures included in it.

The last circumstance function is elaborating in which it realized by *role* type. The *role* showing *guise* is created that can be seen in (n); it has the meaning of *be* applied circumstantially. It is associated with one of the participants in the material clause called Goal (*feedback*) and introduced by preposition *as* to refer to the attribute or identity of the participant.

The results presented in Table 4 were classified based on the process types produced by the male writers. The study has identified several general trends shown by the highest number of the material process. The material clauses were dominated by clauses that represented “doing” if compared to “happening”; for instance, clause (o). Written in the form of a receptive (passive) clause, it indicates a creative clause to convey the outer experience. The verb *found* in the clause signifies a specific transitive clause in which the Goal (*some* problems) as the second participant in the process is the product affected by the process.

**Table 4:** Process type produced by male writers.

No.	Process type	Frequency	Example
1.	Material process	303	*o. Here, in the learning process, some problems are found.* (*Clause M.1.33)*
2.	Verbal process	97	*p. So, the students can speak directly with teacher and friends through this method. (Clause M.1.14)*
3.	Relational process	29	*q. Modelling strategy is giving examples, actions and demonstrations of a certain topic.* (*Clause M.1.28*)
4.	Mental process	23	*r. It is because they feel unnatural to speak in foreign language. (Clause M.1.41)*
5.	Existential process	11	*s. In the teaching learning activities, there is always a model that can be copied. (Clause M.1.27)*
6.	Behavioral process	2	*t. Whilst, the other groups of students as audiences will listen …(Clause M.4.141)*

Besides the material process, the second major process found in this research article category is the verbal process. One notable example is stated in (p); this clause exemplifies a factual verbal action introduced by the verb *speak*. It was performed to state the advantage of a method used in his research to persuade the readers that it is worth-conducted. In this case, the verbal clause is not functioned to project quote or report as its general function; yet it expresses the activity of talking.

The third process is a relational process that is created twenty-nine times in the introduction part. The male writers use two sub-categories of six; firstly, intensive attributive is found in nineteen clauses such as in (q). The process in the clause is attained by the verb *be (is)* to state the meaning of being with two participants involved; first is Carrier (*Modelling strategy*) and second is Attribute (*giving examples, actions…)*. The writer wrote this clause to define the strategy used in his research that intends to give background information to the readers. Secondly, possessive attributive is conveyed in the clause “*Each student has the same chance to speak in the classroom*.” (clause M.1.50). Through the example, the writer shows the meaning of having in which one thing owns another thing. Here, the possessor is labelled to *each student* (Carrier), and *the same chance to speak (Attribute*) is called as the possessed. Moreover, the possession expressed in the clause functions as a process because there are two possibilities; first, the Carrier can be the possessor and the Attribute is the possessed or vice versa.

Positioning the fourth place is a mental process that can be found in twenty-four clauses. The writers use three of four types of mental processes; the first is the cognitive mental process that is used thirteen times. For instance, in the clause “*The teacher must understand whether English in that place is a foreign language or as a second language*.” (Clause M.2.84). The example demonstrates a meta-phenomenal clause in the form of an idea regarding *whether* to introduce the Phenomenon. This clause illustrates the process of obtaining information through thought.

The second is eight perceptive mental processes that deal with the way of regarding something through senses. One of the examples is “*These methods have made students feel bored…”* (Clause M. 5.164). This clause illustrates how students experience some methods stated in the previous clauses through their views indicated by the verb *feel.* Besides two previous types of mental processes, the emotive mental process that is created in two clauses is also found. A striking example of emotive mental process is “*…students were more enthusiastic and enjoyed the activities using two stay two stray.*” (Clause M.5.192). From the example, the writer wants to describe how the students as the Senser denotes an intense feeling about two stay two stray as the technique that would be applied in the research. It aimed at ensuring the readers that this technique has affective benefits to increase students' motivation in learning.

The fifth is the existential process in which it is produced in eleven clauses. To take a concrete example, clause (s) shows the existing thing in the teaching and learning activities. Here, the writer states a kind of phenomenon (*a model*) as the Existent with the verb *be* followed the word *there*. Although this type of clause appears in a few number of clauses, it plays a significant role in introducing the existence of something related to how the writer construes reality through language.

Lastly, the behavioral process is realized in two clauses. One of them is stated in (t); the example signifies a psychological behavior that is represented by the verb *listen*. In this case, the behavioral process interprets the inner experience in mind and realizes it through outer experience in the form of behavior. The Behaver as the only participant involved in the process is represented by a conscious being (*the other group of students*).

Table 5 shows four functions of circumstances element involved in the clauses produced by male writers. Firstly, enhancing function is used in four types: location, manner, cause, and extent. Location as the first type indicating place is utilized as in (u); this example implies absolute spatial information to specify a position in which the verbal process occurs. The second type is manner showing means that is demonstrated in (v). In this example, preposition *by* followed by gerund indicates the instrument providing the way of the material process happens. The next type is presented in (w) that indicates cause signifying reason. Having a sense of *because*, this clause denotes the motive of the relational process takes place. Lastly, the extent showing duration is shown in (x). The temporal duration in the clause functions to give a detailed period given to the second participant in the material process.

**Table 5:** Circumstance type produced by male writers.

No.	Circumstance Function	Circumstance type	Frequency	Example
1.	Enhancing	1) Location	76	*u. Teachers should give a lot of time to students to speak****in the classroom****. (Clause M.1.45)*
		2) Manner	25	*v. …they share with others****by using their mother tongue****, not the target language. (Clause M.1.40)*
		3) Cause	13	*w. English is an important subject at school****due to its role****…(Clause M.4.152)*
		4) Extent	3	*x. Next, teacher gives time to the students****for about 10/15 min****to make scenario. (Clause M.1.64)*
2.	Projecting	1) Matter	47	*y. Students should have competence****in speaking ability****. (Clause M.1.5)*
		2) Angle	10	*z. Therefore,****according to Nunan****(2003:56) there are several principles of teaching …(Clause M.2.83)*
3.	Extending	Accompaniment	13	*aa.****Besides****, a great deal number of students in writing class makes the lecturer lack of time…(Clause M. 4.130)*
4.	Elaborating	Role	9	***bb. As the foreign learner****, they often evaluate their success in learning English…(Clause M. 3.105)*

Projecting is the second circumstantial function operated by the male writers realized in the form of *matter* and *angle*. The first is a *matter* that can be found in forty-seven clauses. One example is in (y); this clause contains information about what is described related to the possessive attributive relational clause. Preposition *in* that is usually used to indicate place is used in this clause to introduce the substance. The second is *angle* denoting source, it is illustrated in (z). Through the example, it can be noticed that the circumstance used by the writer is referring to other' idea. Unlike the typical pattern in which the source comes with the Verbal process, in this case, it appears in the existential process.

The third function is the circumstantial element called extending that is written in the form of *accompaniment*; it appears in thirteen clauses in the introduction part. To take the most striking example, clause (aa) shows the *accompaniment* indicating additive in a material clause. The clause serves to illustrate a positive additive that is used to exemplify the process as two cases. The two cases here refer to the entity as the participant involved in the clause that experiences the same process; one of these participants is denoted circumstantially.

The last circumstantial function is elaborating represented by *role* showing *guise*. This type carries a meaning of *be* that can be understood as an attribute or identity realized in the form of circumstance as stated in clause (bb). In providing the answer of *what as* relating to the *role,* the writer introduces it by using the preposition *as* followed by a noun phrase. It is related to the participant of the material process called the Actor (*they*) since it elaborates the Actor's function.

### 4.2 Discussion

In relating the results with previous studies elaborated in the introduction, the current study identifies three elements of experiential meaning tendency produced by female and male writers in their research article writing. Firstly, the number of process types utilized by both female and male writers reveals a slightly distinctive result. The finding is contrary to Adams & Simmons'(2019) who found that female writers tended to create longer composition comprises of more words spelling, while the finding of this current study shows that male writers have written more process (verbs) compare to female. More process can be the indication of longer composition with the appropriate spelling since the analyzed data for this research have through the data reduction process. Although this comparison has not analyzed significantly, it brings a new perspective in writing productivity of different gender.

The dominant use of the material process in both female and male writers' research article writing provides evidence that there is a similarity in both genders in expressing the action of *doing* and *happening*. However, the pattern of presenting the material clause is different in some cases. Female writers construct more clauses in the *transformative* form that indicate the outcome involved the alteration of aspects in the clause, such as the participant or Goal. While male writers use more *creative* form than females denoted by the use of verbs like *make, produce,* and *create.* This result is predictable as Halliday and Matthiessen (2014, p. 124) point out that “The prototypical form of the ‘outer’ experience is that of actions and events; things happen, and people or other actor do things or make them happen.” The outer experience in the previous statement refers to the writer’ experience toward the world around them. Yet, the domination of material clauses produced by both genders is in contrast with Xuan (2018) who found that the relational process was the major process type used by adolescent students in L2 writing. This difference probably because of the distinctive writing product analyzed in which this current study focused on article writing while Xuan analyzed ‘recommending’ and ‘sharing’ text.

Other evidence also shows that female writers use relational process (intensive attributive) more than male writers. Among three classifications of the dimension in the relational process (intensive attributive), both genders have an almost equal pattern with a little distinctive realization. For example, in the *membership order,* both female and male writers tend to create qualitative attributive realized by an adjective or participial verb as Head of the clause. Secondly, male writers produced few *time-phase inceptive* indicated by the verb *become* from the element of *attribution phase* while female writers used mostly *neutral phase*. Lastly, from the *attribution domain*, it can be interpreted that females mostly used the *semiotic* phase compared to males. Moreover, the major form of the relational process of female writers is displayed in a complex sentence. This result supports the research conducted by Suganob-Nicolau and Sukamto (2016), which showed the domination of simple sentences applied by male writers in their writing while the female writers tended to produce longer sentences.

If female writers produced more relational process (intensive attributive) than males, male writers tend to use more verbal processes compared to female writers. Verbal process in these articles writing functioned to assign source as information and narrate them to support the writers' stance. The result closely matches those obtained by Alkhrisheh et al. (2019) who stated that male writers favored applying *informative* writing style which means they tend to present more detailed features. It can also be seen from the various type of circumstantial elements in the clauses. In the analysis of verbal clause types, all verbal clauses created by both gender belong to indirect reports and most of them are in the form of a proposition.

The mental process also frequently appears in the article writings of both genders. Surprisingly, male writers employed more varied sensing types, especially the use of the emotive mental process which commonly labelled to female. Whereas female writers incline to utilize the cognitive mental process, which showed logic as usually attached to male characteristics. It may be argued that there is an influence of their personal conscious experience in manifesting this trend. A similar result is found in Munalim's study (2017) suggesting that the second participant in the mental clauses known as *phenomenon* reflected the writers' inner world occurrence.

Besides some differences exposed in the previous elaboration, there is a similarity in both female and male writers' three top process types. Even though the order is not exactly the same, the three major process types consist of material process, relational process, and verbal process. This indicates a slightly different point from Halliday and Matthiessen (2014) who states that the main types of process in English transitivity system are the material, mental, and relational processes. The main types refer to the most frequent types used compared to another. While verbal process's finding to be one of major process types is against the result of a study conducted by Xuan (2018) in which the verbal process is considered to be the minor process utilized by the students (writers).

The process types realized in both female and male writing product is also involving the element of circumstances. Generally, the number of circumstantial element types achieved a similar number. A significant difference appeared in the major occurrence of *matter* in female writing and the highest use of *location* indicating the place in writing produced by the male. Even though almost all kinds of circumstantial elements are employed by both genders, *contingency* type that stipulates when the process dependency existed is absent. This may be due to the type of text (research article) and the topic that did not require this type of circumstance.

## 5 Conclusion

The introduction part of research articles produced by a group of female and male writers provides background information that can be categorized into persuasive writing to convince the readers and persuading them to follow the substance of the idea of the research. From meaning-making choice explored in this study, several conclusions can be drawn; firstly, both genders utilized the same dominant type of process namely material process but in an insignificant different technique. On the other hand, the differences are found in the use of verbal process and relational process (intensive attributive). Female writers created a more relational process that has the meaning of attributing compared to males. However, male writers produced a more verbal process that functioned to cite and quote. Besides, male writers outnumbered female writers in using mental process variety. Secondly, in the circumstantial element perspective, the variety of circumstantial functions used by females and males writers were not much different. One notable finding reveals that female created projecting function (*matter*) in the majority, while male writers produced enhancing function (*location shows place*) in the highest frequency.

The study implies that gender difference may give an impact on how the writers express the experiential meaning in the article writing in terms of sub-types of process and circumstantial elements. It contributes to adding the literature on gender and writing that commonly focused on writing quality. Moreover, the study's result can be a reference to understand female and male writers' product from the SFL point of view. Nevertheless, further study needs to be conducted due to the limitation of this study in terms of research method, the research instrument, and the writing product to be analyzed. It is suggested that a more intensive study is necessary to examine the effect of gender difference on the experiential meaning produced in writing and investigate the other factor that may contribute to the different outcome.

## Declarations

### Author contribution statement

Rosi Anjarwati: Conceived and designed the experiments; Performed the experiments; Analyzed and interpreted the data; Contributed reagents, materials, analysis tools or data; Wrote the paper.

Slamet Setiawan: Conceived and designed the experiments; Analyzed and interpreted the data.

Kisyani Laksono: Conceived and designed the experiments; Contributed reagents, materials, analysis tools or data.

### Funding statement

This research did not receive any specific grant from funding agencies in the public, commercial, or not-for-profit sectors.

### Data availability statement

Data will be made available on request.

### Declaration of interests statement

The authors declare no conflict of interest.

### Additional information

No additional information is available for this paper.
